# The Application of a CMR-B-Scalar Sensor for the Investigation of the Electromagnetic Acceleration of Type II Superconductors

**DOI:** 10.3390/s21041293

**Published:** 2021-02-11

**Authors:** Vilius Vertelis, Saulius Balevicius, Voitech Stankevic, Nerija Zurauskiene, Markus Schneider

**Affiliations:** 1 French-German Research Institute of Saint-Louis, 68300 Saint-Louis, France; MARKUS.SCHNEIDER@isl.eu; 2 Center for Physical Sciences and Technology, Department of Functional Materials and Electronics, LT-10257 Vilnius, Lithuania; saulius.balevicius@ftmc.lt (S.B.); voitech.stankevic@ftmc.lt (V.S.); nerija.zurauskiene@ftmc.lt (N.Z.); 3 Faculty of Electronics, Vilnius Gediminas Technical University, LT-03227 Vilnius, Lithuania

**Keywords:** electromagnetic launch, type II superconductors, CMR-B-scalar sensor, magnetic field dynamics

## Abstract

In this paper, we investigated the behavior of a type II superconducting armature when accelerated by a pulsed magnetic field generated by a single-stage pancake coil. While conducting this investigation, we performed a numerical finite element simulation and an experimental study of the magnetic field dynamics at the edge of the pancake coil when the payload was a superconducting disc made from YBa_2_Cu_3_O_7−x_, cooled down to 77 K. The magnetic field measurements were performed using a CMR-B-scalar sensor, which was able to measure the absolute magnitude of the magnetic field and was specifically manufactured in order to increase the sensor’s sensitivity up to 500 mT. It was obtained that type II superconducting armatures can outperform normal metals when the launch conditions are tailored to their electromagnetic properties.

## 1. Introduction

Electromagnetic (EM) launch is a technology which produced a significant science-to-technology breakthrough in the field of solid body acceleration up to space velocities. The list of its possible applications includes space launch [1,2,3], transportation [4,5], material research [6,7,8] and even defense technologies [9,10,11]. A coil accelerator or coilgun, also known as a Gauss gun, is a type of electromagnetic (EM) launcher that accelerates ferromagnetic (reluctance coilgun) or conductive (induction coilgun) armatures to high velocities by employing magnetic fields. The lack of any physical contact between the barrel and the launch package as well as the absence of a propellant eliminates friction and reduces the mass of the launch package, while in turn reducing the amount of energy needed to reach the desired velocities. On the other hand, a complex modular power delivery system with high requirements for switches, real-time projectile tracking and control is required [9,12]. Joule heating, armature flux capture and structural integrity are the factors which limit the performance of metal armatures [13,14].

Type II superconductors are intriguing materials which behave very differently from normal conductors. These superconductors below their transition temperature $T_{c}$ exhibit a near-zero resistivity when the currents through them are below a certain value called a critical current. The resistivity that emerges when the current approaches this critical value is not caused by electron scattering but rather by the motion of quantized magnetic field lines accelerated by the Lorentz force acting on them. Above the critical current, the resistivity of the superconductor increases rapidly, and the superconductor goes back into its normal (resistive) state. This nonlinear behavior makes the electric and magnetic properties of these materials attractive for various applications [15,16,17], one of which is electromagnetic launching. The use of these materials as armatures for electromagnetic launchers can reduce some of the limitations imposed on normal conductors as well as cause some new unforeseen behavior.

At present, only a relatively small number of investigations of the electromagnetic launch of such superconducting armatures have been performed compared to those which investigated the launch of armatures made from normal metals. Calculations performed in [18] demonstrated that a pre-magnetized superconducting armature (using field-cooled magnetization (FCM)) can be accelerated more effectively by a pulsed magnetic field than an armature made from conventional metals. However, this method of acceleration is more complicated when compared to a non-magnetized (NM) superconducting armature, as it adds a preparation stage that requires energy. Another drawback when using a magnetized armature is that it can be necessary to move the armature to the optimal trigger position after the pre-magnetization process [18]. A simple NM armature acceleration method is thus preferable for space applications, such as active removal of space debris by a coilgun mechanism [19]. Unfortunately, up to now, incisive investigations of the electromagnetic launch of NM superconducting armatures have not been conducted.

In the past decade, due to developments in software and computing power, the modelling of the electrodynamic behavior of type II superconductors has become less restrictive, and good agreements between experiments and simulations have been achieved when investigating superconductor magnetization [20,21] and magnetic levitation [22,23,24]. Various eddy current problem formulations have been proposed, suited to handle different superconductor application scenarios or to improve computation times [25,26,27,28,29].

Experimental investigation of the magnetic field behavior when an armature is accelerated by a pulsed magnetic field is still problematic, regardless of the material used. In these setups, the magnetic field is highly inhomogeneous, and its magnitude changes rapidly as does its direction due to the movement of the armature. Thus, for high accuracy measurements, it is necessary to use an especially small, sensitive and fast sensor. These requirements are well met by the CMR-B-scalar sensor [30], which is able to measure the magnitude of the magnetic field independent of its orientation and has been successfully used in investigations of the EM acceleration of a metallic ring [31], cylindrical metallic armatures [32], as well as in studies of magnetic pulse welding of metals [33].

In this paper, we are presenting the results of both experimental and theoretical investigations of the electromagnetic acceleration of an NM armature made from a type II superconductor. For these studies, we used a disc-shaped superconducting armature made from bulk YBa_2_Cu_3_O_7−x_ (YBCO) material, which was launched vertically by a magnetic field generated by a pancake coil. The investigation of the magnetic field dynamics was carried out by numerical finite element modelling (FEM) in combination with the experimental measurements obtained using a unique CMR-B-scalar sensor that was specifically adapted to the experimental conditions. The obtained results are compared with armatures made from high conductivity metals, such as copper and aluminum.

## 2. Experimental Setup

**Figure 1:**
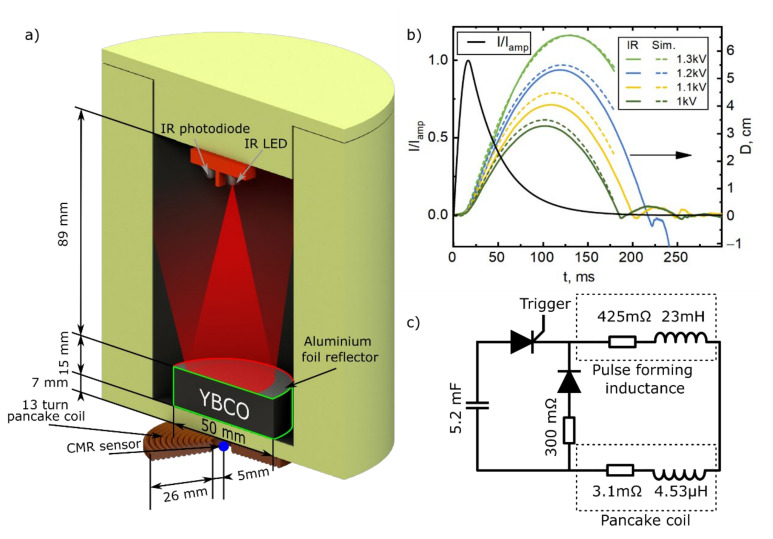
Experimental setup: schematic diagram and geometry of the coilgun (**a**), normalized current pulse produced by the pulse forming unit (black curve) and typical displacement measurements (**b**). An electrical diagram of the pulse forming unit is presented in (**c**).

The electromagnetic acceleration of the superconducting armature was performed in a vertical arrangement. This allowed for easy cooling and did not require any mechanical guides for the armature. Moreover, by measuring the highest altitude reached by the armature and its time of flight, it was possible to easily evaluate the mechanical energy applied to the armature by the electromagnetic acceleration. The schematic diagram of the experimental setup used for these investigations is presented in Figure 1a. The magnetic field source used was a 13-turn pancake coil, made from an enameled copper wire (2 × 4 mm). The coil had an inner diameter of 1 cm, an outer diameter of 6.2 cm and was glued to a textolite housing, which was hollowed out to accommodate the pancake (the housing is not shown in Figure 1a). The distance from the top of the pancake coil to the top of the housing was 2 mm. The isolation gap between coil windings was about 0.1 mm. The container for the liquid nitrogen, which served as a cryostat, was made from polystyrene and was mounted on top of the housing. The base of the cryostat on which the YBCO sample rested had a thickness of 5 mm; thus, the total distance between the surface of pancake coil and the superconducting armature was *l*_cd_ = 7 mm. The cryostat was closed with a lid, leaving an 8.5 cm distance for the projectile to travel vertically inside it.

For the superconductor launch experiments, a commercially available bulk single domain seed melt grown YBCO sample with a critical temperature of ⁓90 K [34] was used. The sample had a disc geometry with a diameter of *d*_D_ = 50 mm, a height of *h*_D_ = 15 mm and a mass (*m*) of 171 g. The disc was cooled down by slowly dripping liquid nitrogen onto it until the nitrogen began to wet the YBCO surface, after which the nitrogen was poured onto it until it was submerged. The experiments were performed once the nitrogen boiled off and was no longer visible at the bottom of the container in order to avoid any drag caused by the residual nitrogen.

Power to the pancake coil was delivered from a pulse forming unit (PFU). A schematic electrical circuit of the PFU is presented in Figure 1c. The PFU generated a single, tooth-shaped current pulse with a ~16 ms rise time and a ~150 ms decay (“tail”) (see black curve in Figure 1b). The PFU capacitor bank was rated for 5 kV charging voltage and was able to produce about a 2.5 T amplitude magnetic field pulse at the center of the pancake coil. The PFU was equipped with a large (23 mH) pulse forming inductance (compared with the 4.53 μH of the pancake coil) in order to reduce the superconductor’s influence on the total inductance of the circuit. This allowed the use of a standard pulse shape, regardless of the amplitude of the pulse or the presence of the superconducting armature. To minimize the influence of the mechanical stress on the superconductor created by the transient magnetic field, the amplitude of the magnetic field pulse used for the EM acceleration was no more than 0.35 T. This magnetic pulse amplitude was able to launch the superconductor to the upper limits of our measuring range.

The CMR-B-scalar sensor used for the pulsed magnetic field measurements was made from a 0.4 μm thick La-Sr-Mn-O (LSMO) film grown onto a polycrystalline Al_2_O_3_ substrate by the pulsed injection metal–organic chemical vapor deposition technique [35]. For the relatively low magnetic field values (up to 0.3 T) used in our experiments, it was necessary to have an extremely sensitive sensor. This was achieved by using the special chemical content of the film, i.e., La_0_._82_Sr_0_._18_Mn_1_._15_O_3_ with Mn excess. According to [36], the magnetoresistance of films with this content at room temperature is about 30% higher compared to films with stoichiometric Mn content. Another specific feature of our experiment was the highly inhomogeneous distribution of the magnetic field in the space between pancake coil and the superconducting armature where the sensor was placed. In such case, the accurate measurement of the local magnetic field requires a sensor with an extremely small active volume. For this reason, we used a CMR-B-scalar sensor with the following dimensions: 400 μm width, a 50 μm gap between electrodes and a 0.4 μm thickness. The effective volume of such sensor, estimated by using its highest dimension as its diameter, was ≈10^−2^ mm^3^.

Manganites (LSMO) below the temperature of the transition from the paramagnetic to the ferromagnetic states (*T*_m_) exhibit remnant magnetization [37]. Therefore, special procedures of calibration were required to avoid memory effects when the sensor was used at cryogenic temperatures [38]. This complicated the use of the CMR-B-scalar sensor in the vicinity of a superconductor cooled down to a liquid nitrogen temperature. To circumvent this, the CMR-B-scalar sensor was placed on the central axis of the pancake coil outside the cold zone, so that only the sensor’s encapsulation touched the base of the cryostat. Thus, the active volume of the sensor was at a 7.5 mm distance from the bottom of the superconducting disc-shaped armature. As the temperature of the CMR-B-scalar sensor did not get much lower than room temperature, the sensor demonstrated no memory effects.

It should be noted that the magnetoresistance effect in such thin manganite films has a very low anisotropy (less than 2%) to the direction of the magnetic field when the value of this field is higher than 1T [39]. However, for lower fields, this anisotropy increases with the decreasing magnitude of the field, and in our case was about ±10%. In order to decrease the measurement error due to this anisotropy, we placed the sensor so that the thin film plane was perpendicular to the symmetry axis of the pancake coil. We estimated that a ten degree change in the field direction produces about a 0.3% measurement deviation from the real value.

For measurements of the vertical displacement of the superconducting armature, an analogue IR distance sensor was placed in the middle of the cryostat lid. This sensor measured the intensity of the reflected infrared light, which is a distance-dependent quantity. The bore of the cryostat was covered with an IR absorbing coating to reduce the background signal of the distance sensor. A reflector made from thin aluminum foil was mounted on the top surface of the YBCO disc to increase the reflectance and in turn, the signal at greater distances.

Our experiments began with the cooling procedure described above. Once the liquid nitrogen had mostly boiled off, the capacitor bank was charged to the desired voltage. After the liquid nitrogen was no longer visible at the bottom of the cryostat, the cryostat was carefully closed with the lid. The estimated disk temperature based on a measurement made using a resistive thermometer Pt-1000 placed at the bottom of the disk was ~76.6 K. Then, the capacitor bank was discharged through the pulse-forming inductance connected in series with the pancake coil. Typical disc trajectories can be seen in the top right of Figure 1b (colored solid curves). Artefacts seen in the trajectory after armature touchdown were caused by a physical rebound from the bottom of the container. After the experiment, the sample was repositioned and resubmerged in nitrogen for a consecutive test

## 3. Experimental Results

Disk displacement (*D*) studies were performed in the free flight regime when the drag force caused by the surrounding nitrogen gas was negligible. To avoid any rotational motion of the disk-shaped superconducting armature that could lead to an unreliable signal from the IR sensor, the disk center, the pancake coil center and the magnetic field sensor were placed along the same axis. The experimental armature displacement vs. time curves are presented in the top right of Figure 1b. They consisted of several peaks, the first of which shows the armature movement due to the electromagnetic acceleration, while the others are the result of the disk bouncing from the bottom of the cryostat. As it can be seen, the front of the first peak exhibits two phases: a relatively slow (delay) altitude change that lasts approximately 15 ms after the beginning of the current pulse and corresponds to the current pulse rise time, and a fast phase, which lasts up to the time instant when the armature reaches its highest altitude. The displacement growth during the delay phase was more abrupt at higher capacitor bank charge voltages. The fast phase of the displacement curve can be well approximated by a parabolic law that is typical for free fall at the surface of the Earth.

**Figure 2:**
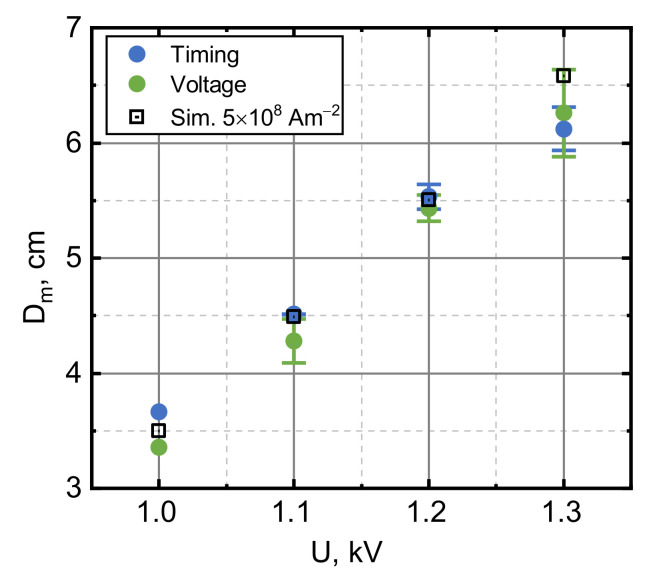
Maximum displacement (*D*_m_) reached by the superconductor for different capacitor voltages. The height calculated from the flight duration is labelled “Timing”. “Voltage” corresponds to the height measured by the sensor, and “Simulation” shows the results obtained from modelling with $j_{C}=5\times 10^{8}{\;\mathrm{Am}}^{-2}$.

Maximum displacements ($D_{m}$) in addition to direct readings of the IR sensor (labelled “Voltage” in Figure 2) were also estimated from the flight durations (labelled “Timing” in Figure 2) as the lift-off and touchdown events could be clearly identified from the displacement curves. The values of $D_{m}$ for different capacitor charge voltages (from 1 to 1.3 kV) are presented in Figure 2. The experimentally used capacitor voltages corresponded to the 450, 490, 530 and 570 A current amplitudes $I_{amp}$ flowing through the pancake coil. At higher charging voltages, the armature rose past the distance sensor calibration range, which covered nearly the whole cryostat. For voltages below 1 kV, the displacement readings were too small to be reliable, and no displacement was observed for voltages below 700 V when the Lorentz force was smaller than the weight of the YBCO disc. “Timing” and “Voltage” approaches gave similar results. $D_{m}$ appeared to follow a nearly linear trend with increasing capacitor voltage within the investigated voltage range.

**Figure 3:**
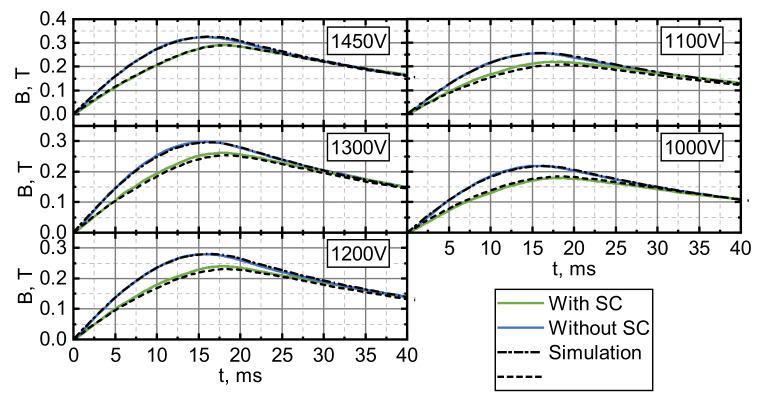
Magnetic field dynamics: the blue curves show the measured field with the empty cryostat. The green curves represent the measurements during the acceleration of the superconductor. The dashed lines are the simulation results of the reference field and the field during superconductor acceleration ($j_{C}=5\times 10^{8}\;{\mathrm{Am}}^{-2}$) for the corresponding capacitor charge voltages.

Figure 3 shows the results of the magnetic field measurements using a CMR sensor placed, as shown in Figure 1a, during experiments with and without the superconductor. In the case of the empty cryostat experiments (labelled “Without SC”), the magnetic field pulses had the same waveforms as the current pulses flowing through the pancake coil. Introducing the superconductor significantly changed the magnetic pulse waveforms coming from the empty cryostat case. The superconducting disk caused a decrease in the magnetic field magnitude. This decrease was strongly expressed in the beginning of the magnetic field pulse and partially covered the pulse decay period. The difference between the reference field magnitude (“Without SC”) and the one during launch (“With SC”) gradually disappeared as the superconducting armature moved away from the pancake coil and its influence on the field in the sensor position diminished. The time until the magnetic field value for “Without SC” and “With SC” became the same, decreased with the increasing capacitor bank charging voltage and was approximately equal to the delay time visible in the displacement vs. time curve (see Figure 1b). This shows that the electromagnetic interaction between coil and superconducting disk mainly took place during this time period.

## 4. Simulation Background

**Figure 4:**
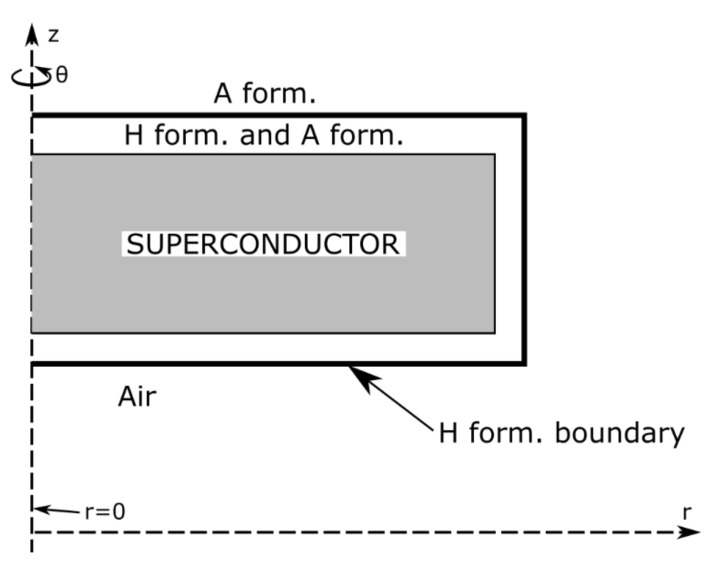
Model geometry: the superconductor domain is represented by the grey square, and the air domain is left white.

The behavior of our experimental setup was also investigated numerically. Figure 4 shows the principal geometry used in our modelling. The dimensions of the superconducting domain used for simulation were the same as that of the sample’s used in the experiments. The nonlinear eddy current problem was solved in the armature’s frame of reference using Maxwell’s equations in their H-formulation (H-form) in 2D axis-symmetric form using Comsol Multiphysics commercial software:

(1) $$\nabla \times E+\mu \frac{\partial H\left(t\right)}{\partial t}=0,$$

(2)  ∇×H=j.

Here, E is the electric field (bold symbols represent vectors), μ—the magnetic permeability, H—the magnetic field and j—the current density. Different E substitution laws were used for the air and superconductor domains. The air was described as having linear resistivity E=ρj, while a power–law relationship was used for the superconducting domain.

(3) $$E=E_{C}{\left(\frac{\left|j\right|}{j_{C}}\right)}^{n}\frac{j}{\left|j\right|}$$

Here, $E_{C}$ is the critical electric field (by convention 1 μV/cm), $j_{C}$ is the current density at which the critical electric field is reached and n is a parameter that describes the “hardness” of the superconductor and models the “steepness” of the superconducting-normal transition. Generally, $j_{C}$ depends on the magnetic field and the temperature. These dependencies were neglected in our model, and a constant $j_{C}$ was assumed. This is physically accurate when the sample does not heat up, and a slowly varying region of the $j_{C}-H$ curve is reached.

The H-form problem is a boundary value problem with a boundary condition [22]:

(4) $$H\left(r,z,t\right)=H_{\mathrm{SC}}+H_{\mathrm{PC}}.\;$$

Here, $H_{\mathrm{SC}}$ is the magnetic field generated by the eddy currents inside the superconductor and $H_{\mathrm{PC}}$ is the field generated by the pancake coil. $H_{\mathrm{PC}}$ is the product of the current pulse $I_{amp}\cdot G\left(d\cdot t\right)$ ($I_{amp}$ is the amplitude of the pulse, G represents the pulse waveform seen in Figure 1b, t is the time and d is the pulse duration multiplier. d = 1 corresponds to the experimental pulse). The magnetic field generated by the coil thus was:

(5) $$H_{\mathrm{PC}}=F\left(r,z+l_{cd}+D\left(t\right)\right)\cdot I_{amp}G\left(d\cdot t\right).$$

Here, the $F\left(r,z\right)$ is the spatial magnetic field map of the pancake coil, which was calculated separately. $H_{\mathrm{SC}}$ was calculated in a separate A formulation module (magnetic fields in AC/DC module) using the current density calculated in the H-form module as an input and treating the entire model domain as air to avoid a secondary generation of eddy currents.

The electrodynamic problem was solved in the superconductor’s frame of reference, offsetting the magnetic field map by the solution of the mechanical problem $D\left(t\right)$ for each time step. The displacement, velocity and acceleration were calculated from the Lorentz force, taking gravity into account. Since the problem is axially symmetric, the dynamic equation for the vertical displacement (D) was:

(6) $$m\frac{d^{2}D\left(t\right)}{dt^{2}}=\int \left(j_{\theta }\cdot \mu H_{r}\right)dV-mg.$$

Here, m is the disk mass (in our calculations 171 g), and $j_{\theta }$ and $H_{r}$ are current density and magnetic field components in θ and r directions. V is the disk volume, and g is the standard gravity (9.8 m/s^2^).

## 5. Simulation Results and Discussion

For the analysis of the experimental results presented in Figure 1, Figure 2 and Figure 3 (*D* vs. *t*, *D*_m_ vs. capacitor bank voltage *U* and *B* vs. *t* at sensors position), we performed calculations using the mathematical procedures presented in Section 4. In our model, the superconductor was described by two parameters: $j_{C}$ and n. The value of the parameter n was chosen to be 20, because at that value, it describes a hard super-conductor with a flux-creep regime [40], which is the most common kind supported by the literature [18,21,41]. The dashed curves in Figure 1b show the modelling results of the *D* vs. *t* dependence. As it can be seen, it well demonstrates all the features of the disc movement obtained experimentally (delay and parabolic phases). Good quantitative agreement (in the frame of experimental error) between the experiments and the calculations was achieved using $j_{C}=5\times 10^{8}\;A/m^{2}$. In order to estimate how reasonable this value was, we calculated the $j_{C}$ using the trapped magnetic field ($B_{t}$ = 1.2−1.3 T) measured at 77 K temperature 1 mm above the superconductor’s surface, which was included in the data sheet provided by the disk manufacturer [34]. This estimation was performed using an analytical formula (7) derived from the Biot–Savart law by its direct integration over a cylindrical domain populated by a constant current density directed azimuthally:

(7) $$j_{C}=\frac{2B_{t}}{\mu }{\left(\left(z-d\right)\ln\left|\frac{\sqrt{a^{2}+{\left(z-d\right)}^{2}}+a}{\left(z-d\right)}\right|-\left(z+d\right)\ln\left|\frac{\sqrt{a^{2}+{\left(z+d\right)}^{2}}+a}{\left(z+d\right)}\right|\right)}^{-1}.$$

Here, $B_{t}$ is the trapped field, a is the radius of the disk, d is its half height and z is the distance between the center of the disk and the point at which the trapped field was measured (on the central axis of the disk). A $j_{C}$ in the range 1.2–1.3 $\times \;10^{8}$ A/m^2^ was obtained. A four times higher $j_{C}$ value that was satisfactory to match our modelling with the experiments can be easily explained by the significantly lower magnetic field (0.35 T) in our experiments than the one generated by the magnetized sample according to the datasheet. Such bulk superconductor $j_{C}$ behavior was provided by the disc manufacturers in [42].

Using fixed $j_{C}$ and n values ($5\times 10^{8}$ $A/m^{2}$ and 20, respectively), the obtained simulations were in good agreement with the experimental data (maximal displacement $D_{m}$ for different U) that is presented in Figure 2. $D_{m}$ was evaluated from the calculated D vs. t curves, taking the peak value. Modelling based on equations presented in Section 4 allowed the simulation of the magnetic field dynamics measured by a CMR-B-scalar sensor (see Figure 3) in both the “Without SC” (dashed–dotted curves) and “With SC” (dashed curves) cases. As it can be seen from Figure 3, the B vs. t dependences measured during the launch of the superconducting disk can be well described by simulations using the same $j_{C}$ and n parameters that were used for the simulation of D vs. t and $D_{m}$ vs. U.

Good agreement between the simulation and the experimental results demonstrated that the modelling method in the armature’s frame of reference, neglecting Joule heating, is suitable for the study of the behavior of type II superconductors during an electromagnetic launch. In the following sections, we present theoretical studies of this configuration, which were outside the limits of our experimental capabilities. We investigated different scenarios with different pancake current waveforms: “step-like” (1 ms rise time) and “experimental” (Figure 1b). In addition, we investigated the effects of critical current density, the armature starting distance from the driving coil and the pulse duration using the experimental current pulse waveform. The performance of the superconducting armatures was compared with Cu and Al armatures of the same geometry, cooled to 77 K.

### 5.1. Influence of j_c_ on Energy Conversion

**Figure 5:**
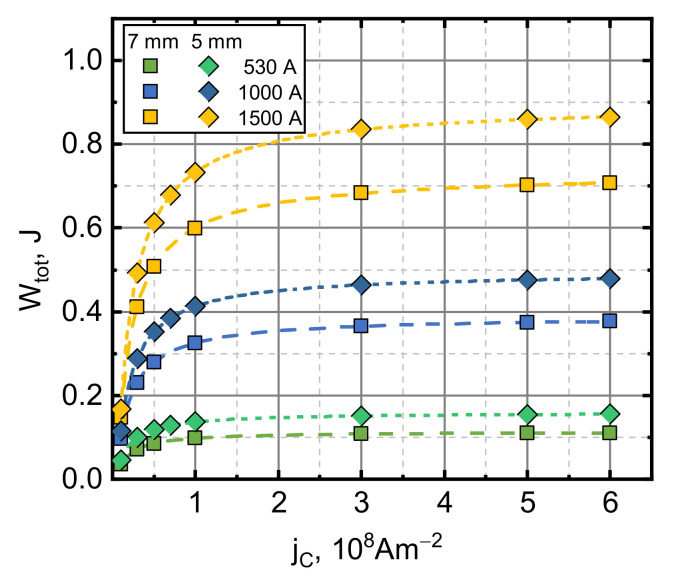
The influence of the critical current density on the total mechanical energy transferred to the superconductor for different current pulse amplitudes and starting heights. The symbols represent the simulation data. The lines are the logistic function fits ($y\left(x\right)=\left(c_{1}-c_{2}\right)/\left(1+{\left(x/x_{0}\right)}^{p}\right)+c_{2}$).

In a vertical acceleration arrangement, the total mechanical energy is the sum of its kinetic energy and potential energy. During acceleration, this energy evolves over time until a steady value $W_{tot}$ is reached. This energy value depends on the electromagnetic energy generated in the pancake coil and the electromagnetic interaction between the superconducting armature and the coil. With a fixed coil current pulse waveform, the amplitude $I_{amp}$ and the armature geometry $W_{tot}$ depends only on the magnitude of $j_{C}$. Calculations of the $j_{C}$ influence on $W_{tot}$ transferred to the armature at different coil current amplitudes were performed using experimental armature geometry at two starting distances from the surface of the pancake coil and the superconducting armature: 7 (our experimental condition) and 5 mm, presented in Figure 5. Initially, as can be seen in Figure 5, an increase in the $j_{C}$ leads to an increase in $W_{tot}$ transferred to the superconductor, but with sufficiently high $j_{C},$ this transfer tends to saturate. This can be explained by the decrease of the magnetic field penetration depth, which becomes shallower and approaches a surface field limit due to the increase in the $j_{C}$. This depth then again increases with the field amplitude and the surface current limit is pushed to higher $j_{C}$ values. As can be seen in Figure 5, under otherwise identical launch conditions (pulse waveform and amplitude), a decrease in the distance between the pancake coil and the superconducting armature $l_{cd}$ increases the $W_{tot}$ value. Lowering the $l_{cd}$ by 2 mm causes increases in the $W_{tot}$ of about 40%, 27% and 22% for pulse amplitudes of 0.53, 1 and 1.5 kA, respectively. These results indicate a way of making more economical launch systems. Relatively high launch efficiency can be reached using a superconductor with a relatively low $j_{C}$. Thus expensive, high $j_{C}$ armature material could be replaced by materials with a lower $j_{C}$ without a significant decrease in the launch efficiency.

### 5.2. Effects of Pulse Amplitude

**Figure 6:**
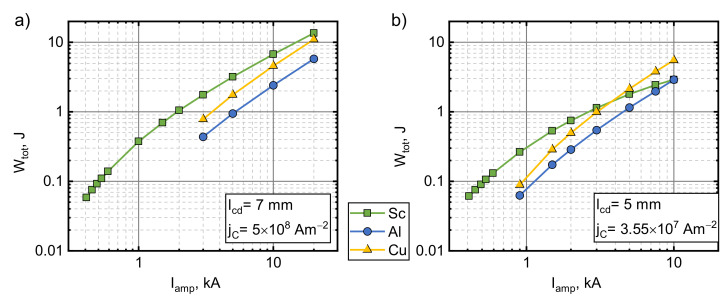
The influence of the current pulse amplitude on the total mechanical energy transferred to an armature made from a type II superconductor, copper or aluminum for the experimental pulse shape and duration. $l_{cd}=7\;\mathrm{mm},\;j_{C}=5\times 10^{8}\;A/m^{2}$ (**a**) and $l_{cd}=5\;\mathrm{mm},\;j_{C}=3.55\times 10^{7}\;A/m^{2}$ (**b**).

To investigate the behavior of an electromagnetic superconductor launch in a broader range, we performed numerical calculations outside the current amplitude range that our experimental setup could withstand. Two sets of $l_{cd}$ and $j_{C}$ were investigated (7 mm with 5 $\times 10^{8}\;A/m^{2}$ and 5 mm with $3.55\times 10^{7}\;A/m^{2}$). The first one corresponded to the experimental conditions. The second set produced values of $W_{tot},$ which were close to those obtained in the experimentally investigated scenarios, only with a 2 mm shorter starting distance $l_{cd}$. This set illustrates the worst case that could be justified by experimental error. The performance of the superconductors was also compared with armatures of the same geometry, which were made from copper and aluminum, the materials most commonly used today. We assumed the temperature of liquid nitrogen for all of the materials to make the comparisons fair. Masses and resistivities of the armatures used in the simulations were m= 264 g and $\rho =2.65\times 10^{-9}\;\Omega m$ for copper and m= 79.5 g and $\rho =4.44\times 10^{-9}\;\Omega m$ for aluminum. The results obtained from the simulations are presented in Figure 6 (points up to 600 A correspond to the experimental values). As can be seen, an increase in the current amplitude resulted in a higher energy being transferred to all the armatures. For the superconducting armatures, this increase is larger in the lower amplitude range ($W_{tot}\propto I_{amp}^{\sim 2}$) and reduces to a nearly linear relationship for the first parameter set and a sublinear one for the second set. Superconducting armatures performed better than the normal metal ones at these lower current pulse amplitudes in both cases. This advantage decreased with the current pulse amplitude, and in the second parameter set case, copper overtook the superconductor at $I_{amp}\approx 3.7\;\mathrm{kA}$. By extrapolating the data, copper is expected to overcome the superconductor at 43 kA in the first parameter set case.

### 5.3. The Influence of Pulse Duration

The energy obtained by the superconducting armature due to electromagnetic acceleration depends on the duration of the coil current pulse. In addition, the launch process is complicated by the large magnetic field gradient through which the armature passes during acceleration. To investigate how $W_{tot}$ depends on pulse duration (d), we performed calculations using pulses with a fixed current pulse amplitude (1.5 kA) and the waveform shown in Figure 1b, scaled in time by a factor of d. d is the relative pulse time duration, and its value represents how many times the pulse duration is shorter. The experimental pulse duration was taken to be d=1.

**Figure 7:**
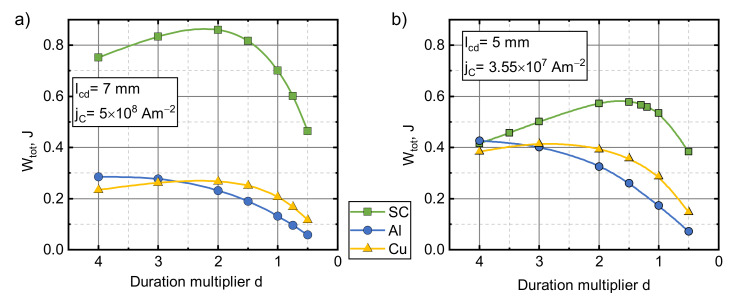
The influence of pulse duration on the total mechanical energy transferred to armatures made from type II superconductor, copper or aluminum. The pulse amplitude was 1. 5 kA. $l_{cd}=7\;\mathrm{mm},\;j_{C}=5\times 10^{8}\;A/m^{2}$ (**a**) and $l_{cd}=5\;\mathrm{mm},\;j_{C}=3.55\times 10^{7}\;A/m^{2}$ (**b**).

Figure 7 illustrates the effects of the d value on $W_{tot}$ in each of the parameter sets. In the case of the first parameter set, the superconductor outperformed the metal armatures within the entire investigated duration range. However, this advantage of the superconducting armature was lower in the case of the second set of values and disappeared when the pulse duration was four times shorter. The metal armatures also showed improvement when brought close to the field source due to the increased magnetic coupling. All of the materials exhibited a peak in their $W_{tot}\;\mathrm{vs}.d$ dependence. The superconductor peaked at a ~2 times shorter pulse for the first set and ~1.5 times shorter for the second one and was ~3 and ~1.5 times more effective than the Cu armature at the same pulse length. Such behavior can be explained by examining both the very long and the very short pulses. At the very short magnetic field pulses, the armature can be considered stationary throughout the pulse. In such cases, the force applied to the armature is proportional to the amplitude of the field for both the metal and superconducting armatures. When the pulse amplitude is kept constant, an increase in the pulse duration acts only by increasing the interaction time between coil and armature, which increases the energy transferred to the armature. However, under the action of a long pulse, the position of the armature cannot be considered static. At a certain moment, the driving force overcomes gravity, and the armature starts to move away from the coil. As it does so, the coupling to the coil decreases and the utilization of the remaining part of the magnetic field pulse for the energy transfer becomes smaller. In the metal armature cases, this increase in the pulse duration also reduces the driving force as the eddy currents decay over time and the field penetrates deeply into the armatures. This explains why metal armatures peaked at higher values of d. The results obtained show that the superconductor can be effectively accelerated using slower magnetic pulses. This opens up possibilities of using cheaper power sources, which do not require fast, high power switching to drive the coil.

### 5.4. Step-Like Field Pulses

**Figure 8:**
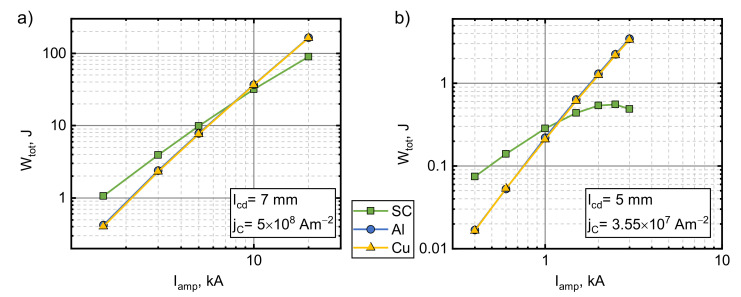
The influence of step-like current pulse amplitude on the total mechanical energy transferred to an armature made from type II superconductor, copper or aluminum. $l_{cd}=7\;\mathrm{mm},\;j_{C}=5\times 10^{8}\;A/m^{2}$ (**a**) and $l_{cd}=5\;\mathrm{mm},\;j_{C}=3.55\times 10^{7}\;A/m^{2}$ (**b**).

The effects of pulse duration showed that the superconducting armature is better than the metal ones at longer pulses. For this reason, we investigated the effects of step-like (rise time of 1 ms) current pulses and their amplitude. This pulse shape has both a high magnetic field transient in the beginning of the pulse and a constant field afterwards. The results for this scenario can be seen in Figure 8. Initially, the energy transferred to the armature increased with the increasing magnitude of the applied field. For the first set of parameters, $W_{tot}$ for the superconductor increased with $I_{amp}$ by a power of 1.7, while for the metals, this power was 2.3. In case of the second set of parameters, the power index of this relationship for metals did not change much and stayed at about 2.5. For the superconducting armature, the initial growth power of ~1.5 decreased with $I_{amp}$ and $W_{tot}$ and began to decrease for the higher values of $I_{amp}$. The superconductor outperformed the metals until the amplitude of the step reached ~7.5 and ~1.15 kA for the first and second parameter sets, respectively. This relatively low current crossover point was in part caused by the strong braking of the superconducting armature (see analysis presented in the next section).

### 5.5. Magnetic Braking

The magnetic braking phenomenon is important for the operation effectiveness of inductive electromagnetic launchers. It is caused by the interaction between an armature and the magnetic field source when the flux that has already penetrated the armature begins to decrease. Magnetic field penetration into the armature is called armature capture [43]. At a certain acceleration stage, it decreases the armature driving force and consequently the efficiency of the synchronous wave induction coilguns, which use metal as the material for the projectile. During electromagnetic acceleration, the flux through an armature can decrease due to the decrease in the external field when the driving current begins to fall or due to its motion in a large magnetic field gradient. When this flux starts to decrease, a counter current is induced. This current then flows in the same direction as the current in the coil, and an attractive force component is introduced. In some cases, this attractive force can overcome the propelling force, and the armature is decelerated.

**Figure 9:**
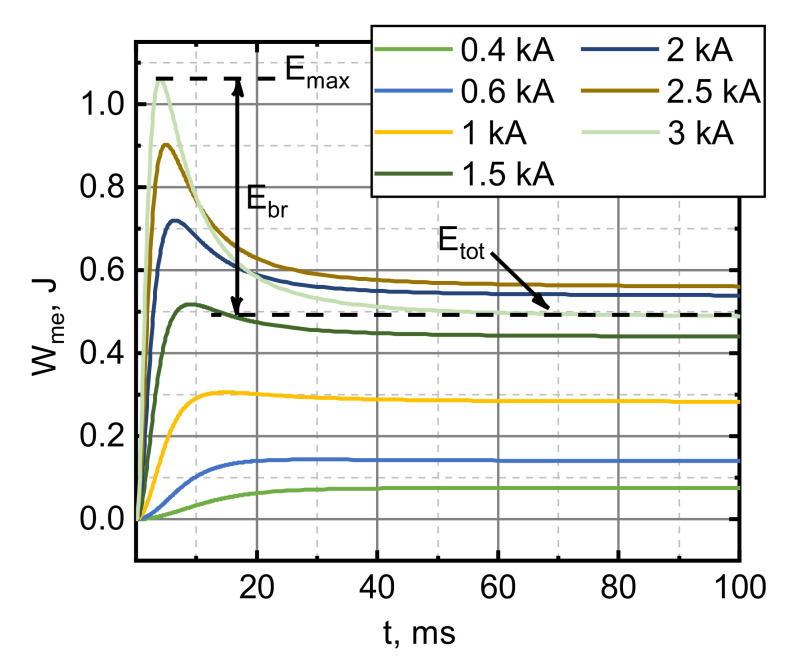
Evolution of the mechanical energy of the superconducting armature over time when the armature was accelerated by a step-like driving current pulse. $l_{cd}=5\;\mathrm{mm},\;j_{C}=3.55\times 10^{7}\;A/m^{2}$.

We obtained that this breaking phenomenon in the case of the superconducting armature is well expressed in the case of step-like pulses. Figure 9 illustrates the evolution of the mechanical energy ($W_{me}\left(t\right)=W_{kinetic}\left(t\right)+W_{potential}\left(t\right)$) over time when a superconducting armature is being accelerated by a step-like pulse, using the second set of parameters. It depicts the transition between no braking and the considerable braking regimes. For low current amplitudes, the $W_{me}$ steadily approaches a constant value ($W_{tot}$). The $W_{me}$ vs. t dependences at higher current amplitudes have a maximum $\left(W_{max}\right)$ that is reached in the beginning of the acceleration, after which they decay to a steady value of $W_{tot}$, which we consider to be the total energy transferred to the armature. The energy lost to magnetic braking or the braking energy is the difference between the $W_{max}$ and $W_{tot}$.

**Figure 10:**
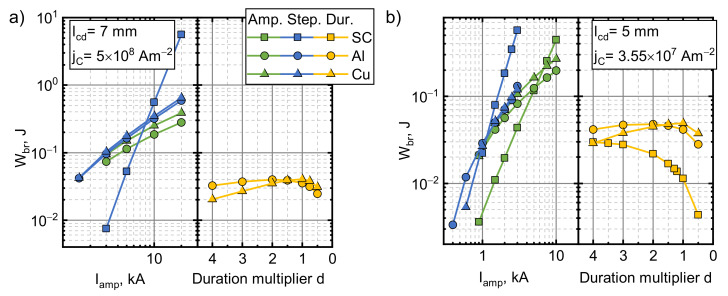
Total mechanical energy lost to magnetic braking. The green line (left) illustrates the effect of the experimental pulse shape amplitude, the blue line, the step-like current pulse amplitude. The yellow line(right) depicts the effects of the pulse duration. $l_{cd}=7\;\mathrm{mm},\;j_{C}=5\times 10^{8}\;A/m^{2}$ (**a**) and $l_{cd}=5\;\mathrm{mm},\;j_{C}=3.55\times 10^{7}\;A/m^{2}$ (**b**).

No magnetic braking was observed in the critical current density parametric sweep. The values of $W_{br}$ for all the other investigated scenarios can be seen in Figure 10. The strongest braking was observed using step-like pulses when $W_{br}$ increased with $I_{amp}$ by the power of ~3.5 and ~3 for the first and second sets, respectively, for the superconducting armature. This strong dependence was the reason why $W_{tot}$ started to decrease with the higher values of $I_{amp}$ when using the second set. Meanwhile, metals showed a nearly linear behaviour. It needs to be noted that even though the power of the relationship was higher for the superconducting armatures, they lost less energy than the metals along a wide range of $I_{amp}$. Magnetic braking could be minimized by rapidly switching the driving current off when the acceleration of the superconducting armature approached zero.

No braking occurred using the first set of $j_{C}$ and $l_{cd}$ for the superconducting armature when the simulations were performed using the experimental pulse waveform. It was, however, observed when using the second set of parameters. In this case, the $W_{br}$ followed a parabolic behaviour ($\propto I_{amp}^{\sim 2}$). Meanwhile, the braking energy for the metals again satisfied a nearly linear trend.

For the pulse duration sweep, the $W_{br}$ of the metal armatures was similar for both starting distances. The superconductor did not show braking with the first set of parameters. The $W_{br}$ seemed to approach a maximum for high d and decreased as d approached 0 with the second set. This behaviour of $W_{br}$ can be explained in the same manner as in the case of $W_{tot}$. At short pulses, the braking energy is mainly dependant on the interaction time and increases with the increasing pulse length. For long pulses, the armature moves away from the coil, thus reducing the magnetic coupling and thereby reducing the energy transfer between the coil and the armature.

**Figure 11:**
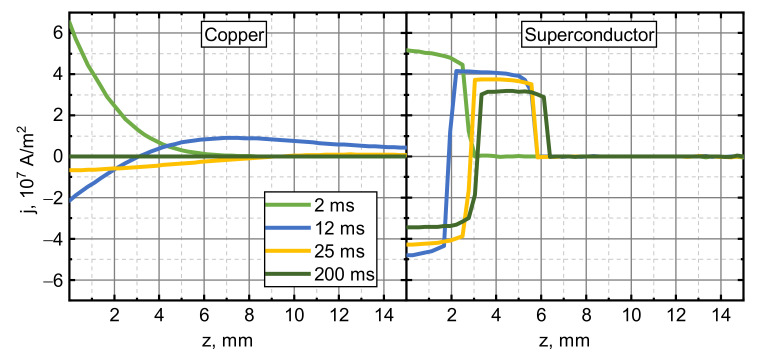
Current density distributions in the copper and superconducting armatures (second set of parameters) along a vertical line 1 cm from the center at different times when accelerated by an experimental current pulse of 3 kA amplitude. $l_{cd}=5\;\mathrm{mm},\;j_{C}=3.55\times 10^{7}\;A/m^{2}$.

Figure 11 shows the induced current density distributions along a vertical line at r = 1 cm at different time instances when armatures were accelerated by an experimental pulse with an amplitude of 3 kA. The current density in the Cu armature (as well as in all other normal conductors) decays both in time and along the line, going deeper into the sample. This results in the current density being strongest near the bottom of the sample, where the magnetic coupling to the driving coil is the strongest. In the superconducting armature case, the current density distribution is rectangular and close to $j_{C}$ in magnitude. A high dB/dt induces an overcritical current density that is highest near the driving coil. This current also decays in time, but the decay rate is negligible below $j_{C}$. The result of this is the generation of two opposite currents circulating within the superconductor, which are located near the surface when the magnetic pulse begins to decrease. For this reason, there is always a non-negligible accelerating force component that mitigates the braking, and superconductors lose less $W_{br}$ than the metals. It follows that larger $W_{br}$ should be expected in situations where the magnetic field penetration depth is large, as the accelerating current would then be situated further away from the coil. This argument agrees well with the braking results discussed earlier (note $W_{br}$ vs. $I_{amp}$ dependences for experimental and step-like current pulses).

Overcritical current densities have to produce substantial Joule heating in the superconductors due to the power-law E−j relationship. For this reason, rapidly changing the magnetic field pulses should be avoided, as it increases the armature temperature and lowers the $j_{C}$. This could damage the armature due to thermal shock. A reasonably slow magnetic field ramp-up to a constant value would mitigate this problem. This field should move synchronously with the superconductor to produce constant acceleration.

**Figure 12:**
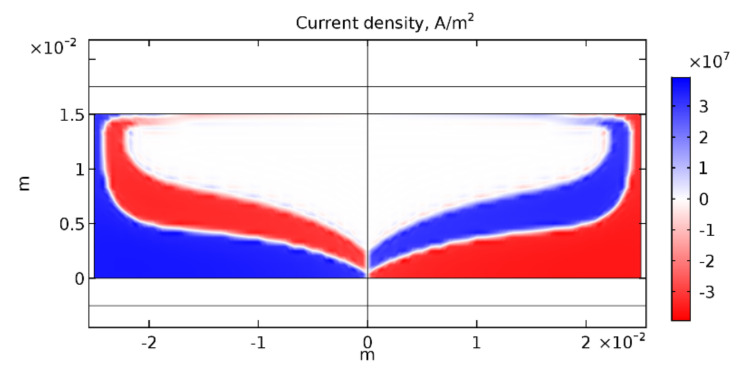
Current density distribution within the superconducting armature for an experimental current pulse with an amplitude $I_{\mathrm{amp}}=1.5$ kA, $j_{C}=3.55\times 10^{7}A/m^{2}$, $l_{cd}=5$ mm at t=0.2 s after the beginning of the pulse.

The nonhomogeneous current distribution in the armature is important not only for the magnetic braking phenomenon but also for the optimal armature geometry design. Figure 12 shows the current density distribution within a superconducting armature ($j_{C}$ = $3.55\times 10^{7}\;A/m^{2}$) 200 ms after a 3 kA experimental pulse. The regions carrying no current only contribute to the total mass of the projectile and are not utilized for its acceleration. The magnetic field did not fully penetrate the superconductor in any of the regimes tested. This suggests that an armature geometry optimization is required to achieve efficient and cost-effective launch setups. The optimized shape will depend on the magnetic field distribution, its amplitude as well as the critical current density of the superconductor.

The examples discussed in this paper illustrate the main differences between normal metals and type II superconductors in coil accelerator applications. When normal metals are used, the induced current density is proportional to the magnetic field derivative and the resistivity of the material. Due to resistive losses, the field diffuses into the normal metals. When superconductors are used, the eddy currents are limited near the value of $j_{C}$ by the highly nonlinear E−j relationship (Equation (3)). For these reasons, these two kinds of materials behave differently in coilgun scenarios. Normal metals require the transient magnetic field to be accelerated, while superconductors can be accelerated by a time constant field (provided it has a gradient). This modifies the requirements for coilgun design and the design of its power supply. A slow field ramp-up is desired to avoid armature heating. A magnetic field wave of a constant amplitude travelling synchronously with the superconducting armature would provide constant acceleration without a time limit in contrast to normal metals where the acceleration decreases due to the armature capture.

## 6. Conclusions

In this paper, we investigated the behavior of an electromagnetically accelerated disk-shaped superconducting YBCO armature cooled to 77 K. The acceleration was performed in a vertical arrangement using a single-stage pancake coil. The vertical displacement of the disk was measured using an IR distance sensor, and the magnetic field was measured using a CMR-B-Scalar sensor with small dimensions, which was able to measure the local magnitude of the magnetic field independent of its direction. It was observed that the motion and the magnetic field dynamics agreed well with the numerical simulations based on Maxwell’s equations in their H-formulation, assuming that the superconductor is a nonlinear conductor with a power-law E−j relationship using realistic values of $j_{C}$. The numerical investigations showed that a higher critical current density of the superconductor increased the mechanical energy transferred to the superconducting armature. This energy, however, had a limit for a given accelerating coil current pulse and armature starting position. The performance of the superconducting armature was numerically compared to normal metal armatures, cooled to liquid nitrogen temperature. This comparison showed that an increase in the current amplitude resulted in higher energy being transferred to all these armatures. For the superconducting armature, this increase was larger in the lower amplitude range and then was reduced to a nearly linear or sublinear relationship depending on the parameter set used for the calculations. Thus, the use of superconducting armatures was advantageous in the lower pulse amplitude range. Investigations of the influence of the pulse duration performed using pulses with a fixed current pulse amplitude and an experimental waveform demonstrated that the superconductor outperforms normal metals when accelerated by magnetic pulses with smaller time derivatives. Simulations using a step-like driving current pulse also revealed that superconductors again outperform normal metals using lower current amplitudes. The range in which they did so was, however, reduced by the strong magnetic braking caused by persistent currents. The mechanical energy lost to the magnetic braking had the strongest dependence on the pulse amplitude for step-like pulses, where the interaction was not mitigated by the reduction in the driving current. As eddy currents in type II superconductors do not decay past the critical current density, the use of superconducting armatures reduces the armature capture limitation and can increase the effectiveness of synchronous induction coilgun launchers.
